# Selected using bioinformatics and molecular docking analyses, PHA-793887 is effective against osteosarcoma

**DOI:** 10.18632/aging.203165

**Published:** 2021-06-21

**Authors:** Bo Wu, Wenzhuo Yang, Zhaoyu Fu, Haoqun Xie, Zhen Guo, Daqun Liu, Junliang Ge, Sheng Zhong, Luwei Liu, Jingyi Liu, Dong Zhu

**Affiliations:** 1 Department of Orthopaedics, The First Hospital of Jilin University, Changchun, China; 2 Clinical College, Jilin University, Changchun, China; 3 Department of Liver and Gallbladder Surgery, The First Hospital of Jilin University, Changchun, China; 4 Department of Neurosurgery, Cancer Hospital of Sun Yat-sen University, Guangzhou, Guangdong, China; 5 Department of Biomedical Informatics, Harvard Medical School, Boston, MA 02115, USA

**Keywords:** bioinformatics, drug treatment, osteosarcoma, bone science, prognosis

## Abstract

To identify novel prognostic and therapeutic targets for osteosarcoma patients, we compared the gene expression profiles of osteosarcoma and control tissues from the GSE42352 dataset in the Gene Expression Omnibus. Differentially expressed genes were subjected to Gene Ontology, Kyoto Encyclopedia of Genes and Genomes, Gene Set Enrichment and protein-protein interaction network analyses. Survival curve analyses indicated that osteosarcoma patients with lower mRNA levels of cyclin-dependent kinase 1 (*CDK1*) and topoisomerase II alpha had better prognoses. Various computer-aided techniques were used to identify potential CDK1 inhibitors for osteosarcoma patients, and PHA-793887 was predicted to be a safe drug with a high binding affinity for CDK1. *In vitro*, MTT and colony formation assays demonstrated that PHA-793887 reduced the viability and clonogenicity of osteosarcoma cells, while a scratch assay suggested that PHA-793887 impaired the migration of these cells. Flow cytometry experiments revealed that PHA-793887 dose-dependently induced apoptosis in osteosarcoma cells. Western blotting and enzyme-linked immunosorbent assays indicated that CDK1 expression in osteosarcoma cells declined with increasing PHA-793887 concentrations. These results suggest that PHA-793887 could be a promising new treatment for osteosarcoma.

## INTRODUCTION

Osteosarcoma is a primary malignant bone tumor originating from osteochymal cells. During osteosarcoma, proliferating tumor cells directly form immature bone or osteoid tissue. The most common sites of osteosarcoma are long bones such as the distal femur, proximal tibia and humerus, while other sites are rare [1]. Hematogenous metastasis occurs early and frequently in osteosarcoma patients, and tends to progress rapidly. Before 1970, the standard treatment for osteosarcoma was amputation, but 80% of patients had minimal metastases at the time of diagnosis, and the average time from surgical treatment to pulmonary metastasis was eight months. The five-year overall survival rate was low, and most patients died within a year of their diagnosis [2]. In recent years, with the development of chemotherapeutic drugs, surgical techniques, bone reconstruction methods and other treatment options, osteosarcoma limb salvage has gradually replaced amputation. Limb salvage is now the first choice for over 80% of patients, and the five-year overall survival rate has increased from about 20% to 55–75% [3]. However, these statistics are still unsatisfactory for both patients and surgeons.

Recently, bioinformatic methods and microarray technologies have been increasingly used to determine the genetic alterations and molecular pathways involved in the initiation, progression and metastasis of osteosarcoma. Zhang et al. [4] demonstrated that the upregulation of solute carrier family 2, facilitated glucose transporter member 11 and chromogranin B may be important for osteosarcoma progression. Wang et al. [5] also indicated that the transforming growth factor β1 system may contribute to osteosarcoma progression. However, further studies on the molecular mechanisms of osteosarcoma are urgently needed.

Cyclin-dependent kinase 1 (CDK1) belongs to the family of serine/threonine protein kinases, and is a key cell cycle regulator [6]. CDK1 is necessary for the proliferation of mammalian cells, and is the only CDK that can initiate mitosis (M phase) [7]. Tumor cells often exhibit abnormal proliferation, overexpression or overactivation of cyclins, reduced CDK inhibitor activity, continuous activation of upstream mitotic signals, and other alterations that ultimately impact CDK activity [8]. Because CDK activity is necessary for cell division and is often enhanced in tumor cells, the identification of effective CDK1 inhibitors is an important step in drug development and cancer treatment.

Virtual screening and molecular docking are widely used methods in rational drug design and pharmaceutical chemistry [9–10]. These techniques can be used to determine the binding affinities between proteins and ligands at the atomic level, as well as to calculate various pharmacological properties of specific ligands [11–12]. In the present study, we combined bioinformatics with virtual screening analyses to accelerate drug discovery for osteosarcoma. First, we used a public dataset to identify differentially expressed genes (DEGs) between osteosarcoma and normal tissues. Subsequently, we explored the functions of these DEGs and constructed a protein-protein interaction (PPI) network. We then validated the expression of the network hub genes and assessed the correlation between hub gene levels and osteosarcoma patients’ prognoses. Finally, we used molecular docking methods to identify a small molecule inhibitor of CDK1, and conducted cytological experiments to determine its potential as an anti-osteosarcoma drug. This study has provided a novel medication candidate for osteosarcoma treatment.

## RESULTS

### Identification of DEGs

**Figure 1:**
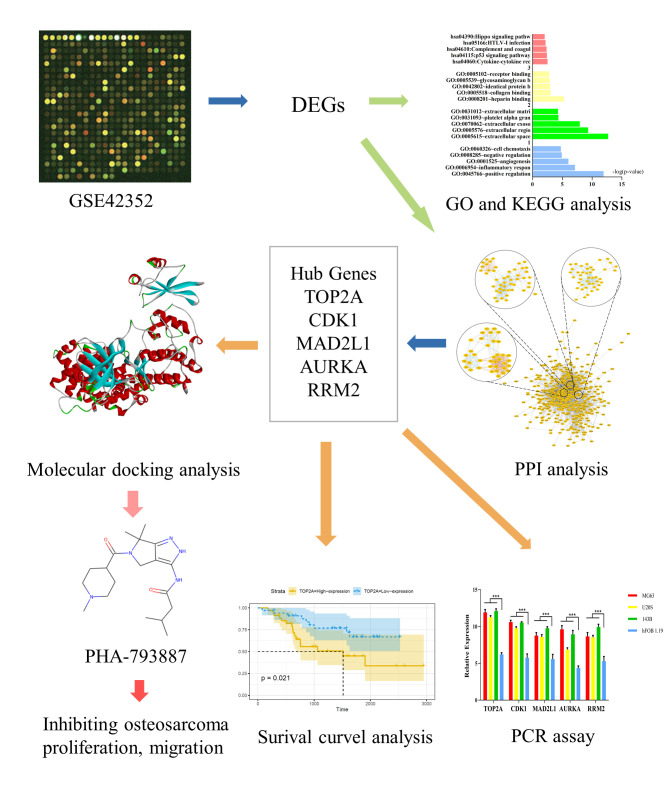
**Study framework.** The first image was selected to represent the tissue datasets from the Gene Expression Omnibus database.

**Figure 2:**
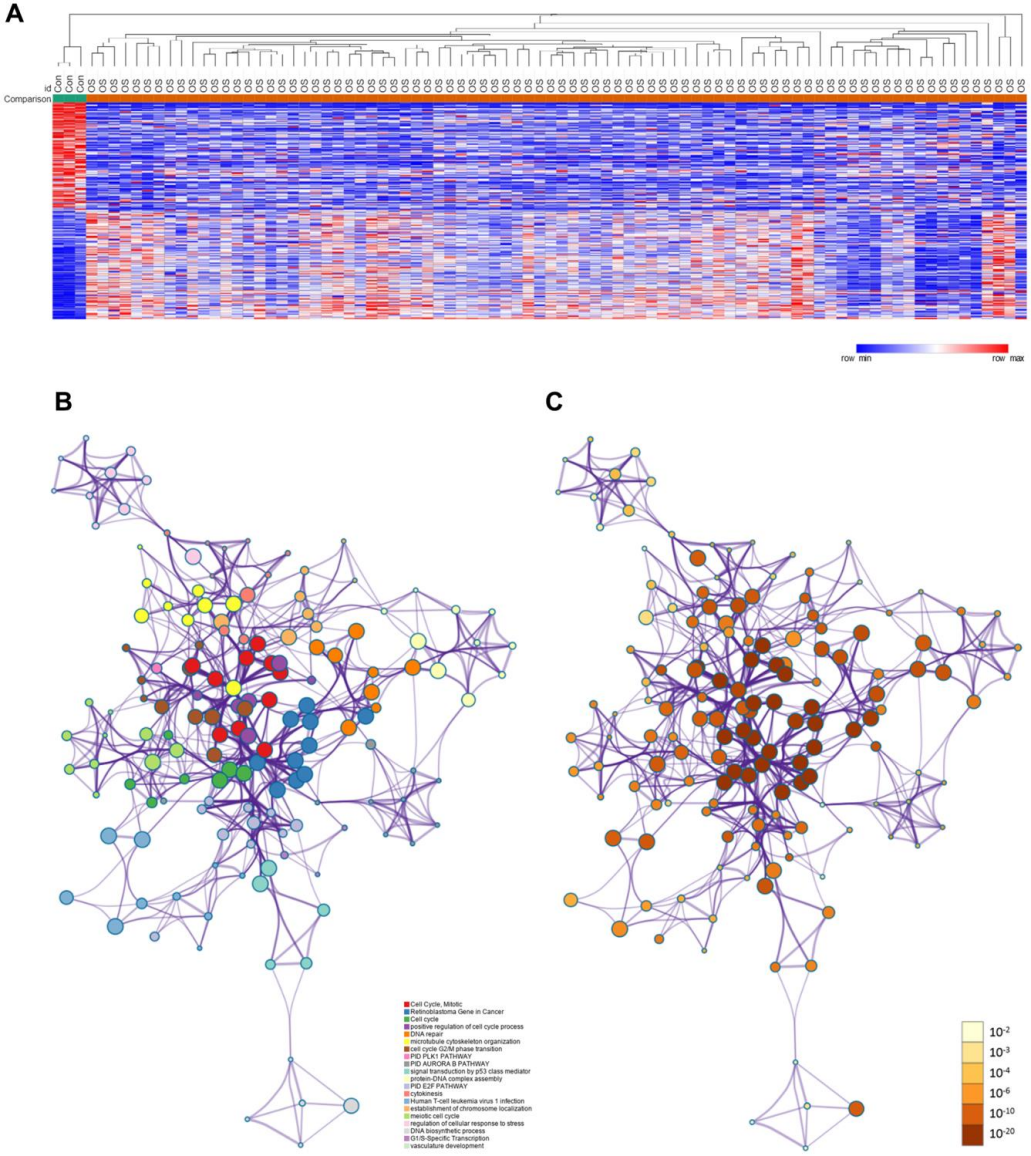
**DEGs between osteosarcoma and normal tissues.** (**A**) Heat map of the DEGs in GSE42352. (**B**) DEGs colored by cluster ID. DEGs in the same cluster ID nodes are closely related to each other. (**C**) DEGs colored by *P*-value. Terms with more significant *P*-values contain more genes.

The framework of this study is shown in Figure 1. First, we used the GSE42352 dataset to identify DEGs between osteosarcoma and normal tissues. In total, 543 DEGs were detected, including 280 upregulated and 263 downregulated genes. Heat maps of DEG expression are shown in Figure 2A. In Figure 2B and 2C, each colored node represents the cluster ID and *P*-value for an enriched term.

### Functional and pathway enrichment analyses

Next, we used Metascape to perform Gene Ontology (GO) and Kyoto Encyclopedia of Genes and Genomes (KEGG) pathway enrichment analyses on the DEGs. The DEGs were mainly enriched in the ‘cell cycle’, ‘positive regulation of cell cycle process’, ‘DNA repair’, ‘microtubule cytoskeleton organization’ and ‘cytokinesis’ (Supplementary Figure 1A).

We also performed GO and KEGG pathway enrichment analyses using the Database for Annotation, Visualization and Integrated Discovery (DAVID). The results from the GO biological process analysis indicated that the upregulated genes in osteosarcoma patients were significantly enriched in ‘positive regulation of angiogenesis’, ‘inflammatory response’ and ‘angiogenesis’. The GO molecular function analysis revealed that the upregulated genes were mainly involved in ‘heparin binding’, ‘collagen binding’ and ‘identical protein binding’. The GO cellular component analysis demonstrated that the upregulated genes were enriched in the ‘extracellular space’, ‘extracellular region’ and ‘extracellular exosome’. The downregulated genes were enriched in the same functions. The results of the KEGG pathway enrichment analysis indicated that the upregulated and downregulated DEGs were enriched in ‘cytokine-cytokine receptor interaction’, ‘p53 signaling pathway’, ‘complement and coagulation cascades’ and ‘HTLV-I infection’ (Supplementary Figure 1B and 1C, Supplementary Table 1).

A Gene Set Enrichment Analysis (GSEA) was also employed to identify abnormally regulated pathways in osteosarcoma patients. The results revealed that the mammalian target of rapamycin signaling pathway and autophagy were significantly enriched in osteosarcoma patients (Supplementary Figure 1D and 1E).

### PPI network construction and module selection

We then used the Search Tool for the Retrieval of Interacting Genes (STRING) to construct a PPI network from the DEGs. Using Cytoscape software, we identified 18 hub genes with degree values ≥ 100: *TOP2A, CDK1, CDC20, MAD2L1, CCNA2, RFC4, CDKN3, AURKA, AURKB, RRM2, CCNB2, CENPA, FEN1, TYMS, CDC45, BUB1, MCM4* and *MCM2* (Table 1). Hub gene expression was then verified using the GSE14359 dataset, and the results were in agreement with those of the GSE42352 dataset (Supplementary Figure 2).

**Table 1:** Detailed information about the hub genes.

**Gene symbol**	**Degree**	**Betweenness Centrality**	**Gene symbol**	**Degree**	**Betweenness Centrality**
TOP2A	131	0.12491649	RRM2	107	0.0238615
CDK1	128	0.04926424	CCNB2	106	0.0133571
CDC20	111	0.02655093	FEN1	105	0.0114579
MAD2L1	110	0.01919526	CENPA	105	0.01718783
CCNA2	110	0.00594959	TYMS	104	0.01483465
RFC4	109	0.0092881	CDC45	103	0.00353462
CDKN3	108	0.01945569	BUB1	102	0.00773264
AURKA	108	0.02507401	MCM4	101	0.00264547
AURKB	107	0.00713131	MCM2	100	0.00314447

**Figure 3:**
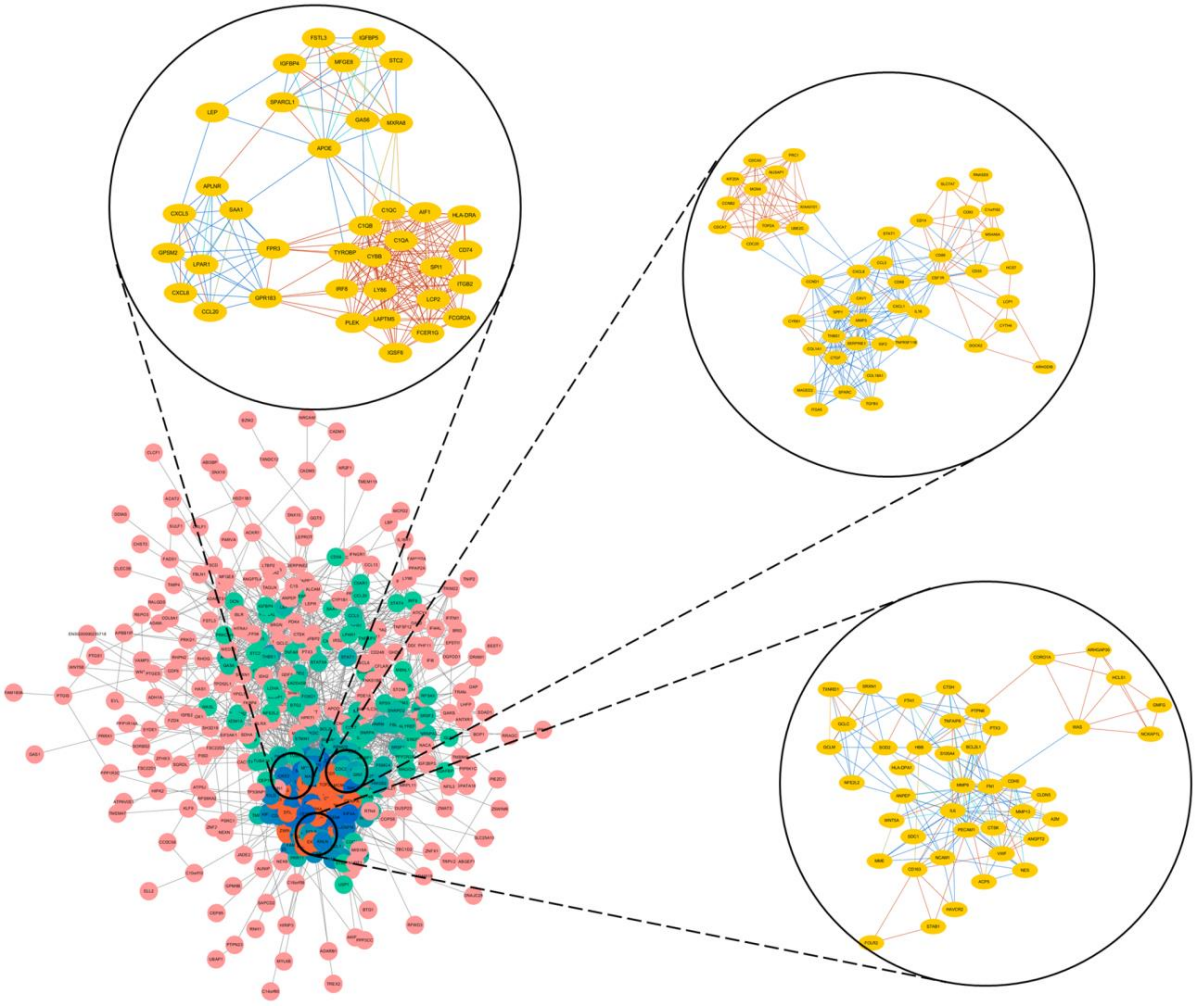
Top three modules from the PPI network.

Next, we selected the three most significant modules from the PPI network, and functionally annotated the genes in the modules (Supplementary Table 2, Figure 3). An enrichment analysis demonstrated that the genes in module 1 were mainly associated with ‘cell division’, ‘mitotic nuclear division’ and ‘DNA replication’. The genes in module 2 were involved in ‘mRNA splicing, via spliceosome’, ‘spliceosome’ and ‘catalytic step 2 spliceosome’. The genes in module 3 were mainly enriched in ‘platelet degranulation’, ‘platelet alpha granule lumen’ and ‘positive regulation of protein kinase B signaling’.

### Measurement of hub gene expression using quantitative real-time PCR (qRT-PCR)

**Figure 4:**
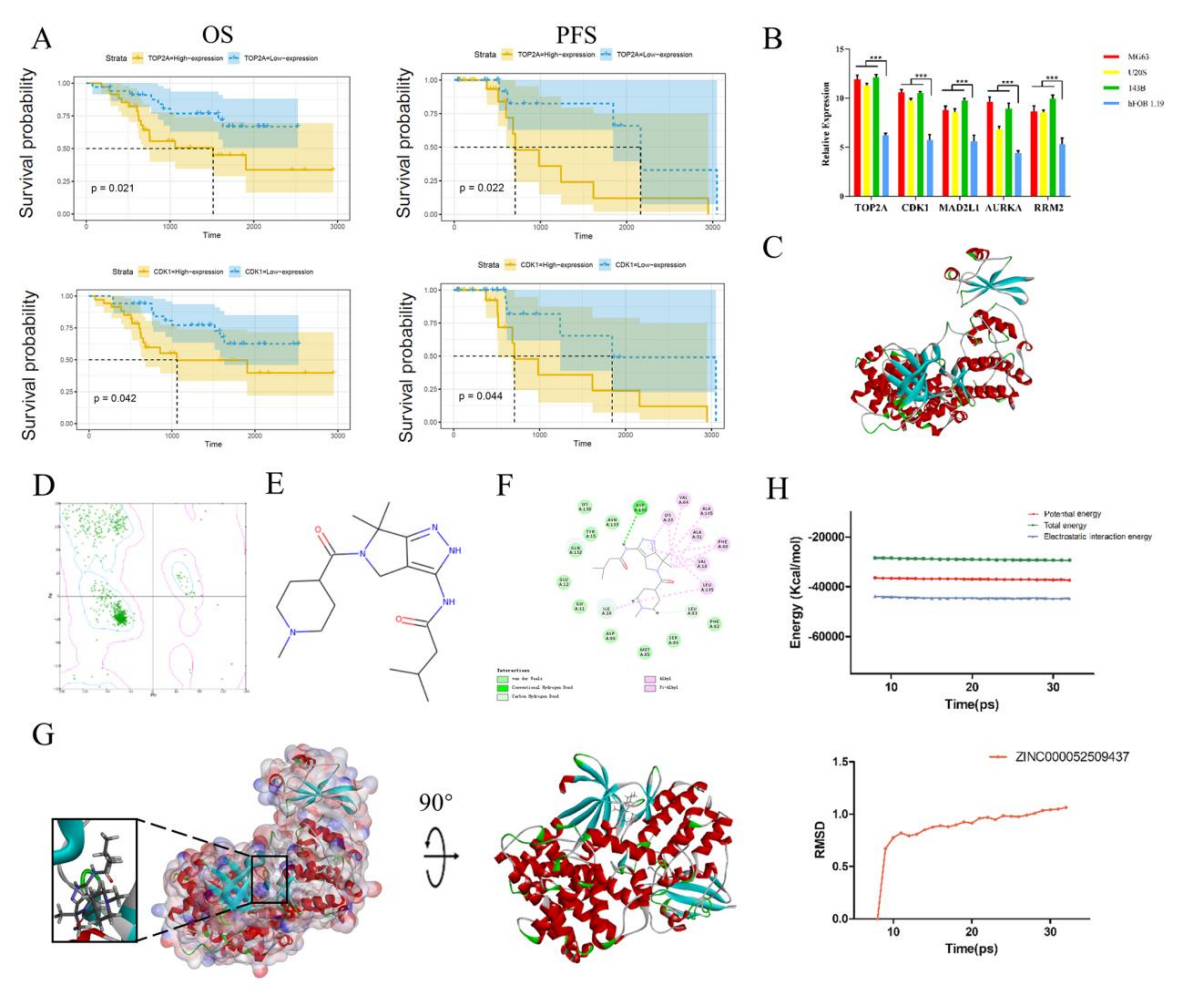
**Survival curve analysis, hub gene validation and molecular docking analysis.** (**A**) Kaplan-Meier estimates of PFS and OS in osteosarcoma patients based on *CDK1* and *TOP2A* expression. (**B**) Validation of *TOP2A*, *CDK1*, *AURKA*, *MAD2L1* and *RRM2* expression *in vitro*. ^*^P < 0.05. (**C**) Crystal structure of CDK1. (**D**) Ramachandran diagrams of CDK1. (**E**) Structure of PHA-793887. (**F**) 2D intermolecular interaction diagram of the PHA-793887/CDK1 complex. (**G**) Schematic drawing of the interactions between CDK1 and PHA-793887. (**H**) Potential energy profile and root-mean-square deviation curve of the PHA-793887/CDK1 complex obtained from the molecular dynamics simulation.

Subsequently, we performed qRT-PCR to measure *TOP2A, CDK1, MAD2L1, AURKA* and *RRM2* expression in human normal osteoblast cells (hFOB 1.19) and osteosarcoma cells (MG63, U208 and 143B). These genes were all expressed at higher levels in osteosarcoma cells than in normal osteoblast cells (*P* < 0.05), as shown in Figure 4B.

### Survival curve analysis based on hub gene expression

We then performed a survival curve analysis to determine whether *TOP2A* or *CDK1* levels were associated with osteosarcoma patients’ prognoses in the Therapeutically Applicable Research to Generate Effective Treatments dataset. Osteosarcoma patients with lower *TOP2A* levels exhibited significantly longer overall survival (OS) and progression-free survival (PFS) than those with higher *TOP2A* levels (*P* < 0.01) (Figure 4A). Likewise, osteosarcoma patients with lower *CDK1* levels exhibited significantly longer OS and PFS than those with higher *CDK1* levels (*P* < 0.01). These results indicated that higher *TOP2A* and *CDK1* levels are associated with a poorer prognosis in osteosarcoma patients (Figure 4A).

### Absorption, distribution, metabolism, excretion (ADME) and toxicity properties of CDK1 inhibitors

Since *CDK1* was upregulated in osteosarcoma tissues/cells and associated with a poorer prognosis, we downloaded 20 CDK1-related drugs from the ZINC15 database and assessed their ADME and toxicity properties. Among these compounds, ZINC000052509437 (PHA-793887) was identified as an ideal, effective and safe CDK1 inhibitor (Table 2). PHA-793887 had excellent aqueous solubility (water temperature: 25°C, Score: 3) and a plasma protein binding property (Score: 1). Moreover, PHA-793887 was found to be safe based on a hepatotoxicity evaluation, National Toxicology Program evaluation, Ames mutagenicity evaluation and developmental toxicity potential property prediction (Table 3). These pharmacological properties should be carefully considered when evaluating CDK1-targeted drug candidates. Therefore, PHA-793887 was selected for further research.

**Table 2:** ADME properties of the top 20 compounds.

**Number**	**Compounds**	**Solubility Level**	**BBB Level**	**CYP2D6**	**Hepatotoxicity**	**Absorption Level**	**PPB Level**
1	ZINC000000023894	2	3	0	1	0	1
2	ZINC000001639355	2	1	0	1	0	1
3	ZINC000002568154	4	2	0	0	0	1
4	ZINC000003814479	2	4	0	1	1	0
5	ZINC000003924157	2	1	0	1	0	1
6	ZINC000003937395	2	3	1	0	0	1
7	ZINC000003938688	2	4	0	1	2	1
8	ZINC000013983251	2	4	0	1	0	1
9	ZINC000014806879	3	3	0	1	0	0
10	ZINC000016052857	3	3	0	1	0	1
11	ZINC000021288966	2	3	1	1	0	1
12	ZINC000028821265	2	1	0	1	0	1
13	ZINC000034894449	3	3	0	1	0	0
14	ZINC000040442496	2	3	0	1	0	0
15	ZINC000043128366	2	4	0	0	0	1
16	ZINC000043131434	3	4	0	1	0	1
17	ZINC000052509437	3	3	0	0	0	1
18	ZINC000053119602	2	2	0	1	0	0
19	ZINC000225710809	2	1	0	1	0	0
20	ZINC000261187328	2	4	0	1	0	1

Abbreviations: BBB: blood-brain barrier; CYP2D6: cytochrome P-450 2D6; PPB: plasma protein binding.

Abbreviations: Aqueous-solubility level: 0: extremely low; 1: very low, but possible; 2: low; 3: good.

Abbreviations: BBB level: 0: very high penetrant; 1: high; 2: medium; 3: low; 4: undefined.

Abbreviations: CYP2D6 level: 0: noninhibitor; 1: inhibitor.

Abbreviations: Hepatotoxicity: 0: nontoxic; 1: toxic.

Abbreviations: Human-intestinal absorption level: 0: good; 1: moderate; 2: poor; 3: very poor.

Abbreviations: PPB: 0: absorbent weak; 1: absorbent strong.

**Table 3:** Toxicity of the top 20 compounds.

**Number**	**Compounds**	**Mouse NTP**		**Rat NTP**		**Ames**	**DTP**
		**Female**	**Male**	**Female**	**Male**		
1	ZINC000000023894	0	1	0.077	0.382	1	1
2	ZINC000001639355	0	0.151	0	0.025	0.014	1
3	ZINC000002568154	0.002	0	1	1	0	0.005
4	ZINC000003814479	0.001	0.953	1	1	0.008	0
5	ZINC000003924157	1	1	0	0.442	0	0
6	ZINC000003937395	0	0.998	0	0.024	0.979	1
7	ZINC000003938688	1	0	0	0.002	0	0
8	ZINC000013983251	1	1	0	0	0	0.03
9	ZINC000014806879	1	1	0	0	0	0.992
10	ZINC000016052857	0.841	0.003	0	0	0	1
11	ZINC000021288966	0	1	0	0.955	1	1
12	ZINC000028821265	0.593	0.984	0	0	1	0.007
13	ZINC000034894449	1	0	1	0	0	1
14	ZINC000040442496	0	0.058	0	0.003	0.449	0.018
15	ZINC000043128366	1	1	0	0.996	0	1
16	ZINC000043131434	0.997	0.009	0	0	0	1
17	ZINC000052509437	0	0	0.791	1	0	0
18	ZINC000053119602	0	1	0.001	0.884	0	0.991
19	ZINC000225710809	0	0.127	0	0.967	0	0.001
20	ZINC000261187328	0.252	0	0	0.002	0	0.055

Abbreviations: NTP: U.S. National Toxicology Program; DTP: developmental toxicity potential.

NTP<0.3(noncarcinogen);>0.8(carcinogen).

Ames<0.3(nonmutagen);>0.8(mutagen).

DTP<0.3(nontoxic);>0.8(toxic).

### Ligand-binding site analysis

Next, we assessed the binding between PHA-793887 and CDK1. A Ramachandran plot indicated that the crystal structure of CDK1 employed in this study (Figure 4C) was stable and obeyed theoretical predication (Figure 4D). Figure 4E shows the structure of PHA-793887. The molecular structure of the PHA-793887/CDK1 complex was generated using the CDOCKER module of Discovery Studio 4.5, and its CDOCKER interaction energy was found to be -50.1452 kcal/mol. Figure 4G shows the interaction between CDK1 and PHA-793887. A structural computation study indicated that PHA-793887 formed three pairs of hydrogen bonds and 12 π-interactions with CDK1 (Supplementary Table 3, Figure 4F). These results demonstrated that the PHA-793887/CDK1 complex is highly stable.

### Molecular dynamics simulation of the PHA-793887/CDK1 complex

We then conducted a molecular dynamics simulation to evaluate the stability of the PHA-793887/CDK1 complex under dynamic conditions. The initial conformations were obtained from the CDOCKER molecular docking experiment. The root-mean-square deviation curves and potential energy of the PHA-793887/CDK1 complex revealed that the complex trajectory reached equilibrium after 20 ps, and the potential energy of the complex stabilized over time (Figure 4H). Thus, the molecular dynamics simulation indicated that PHA-793887 binds steadily to CDK1.

### PHA-793887 reduces the proliferation of osteosarcoma cells

**Figure 5:**
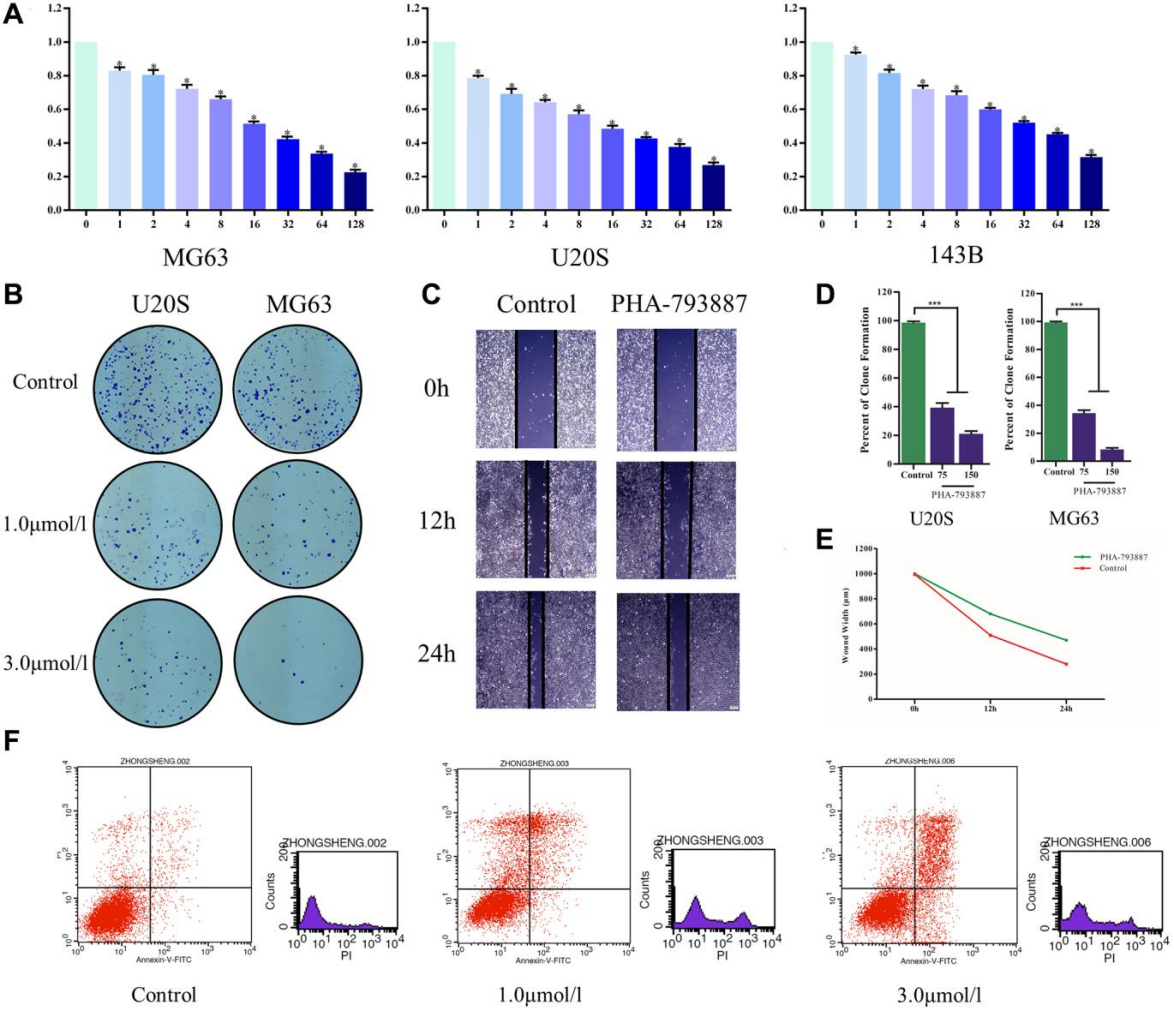
**Anti-osteosarcoma effects of PHA-793887.** (**A**) Cellular viability of osteosarcoma cells treated with PHA-793887. (**B**) Colony formation assay results demonstrating the anti-proliferative effects of PHA-793887 in MG63 and U20S cells. (**C**) Scratch assay results demonstrating that PHA-793887 suppressed the migration of osteosarcoma cells. (**D**) Numbers of clones formed by MG64 and U20S cells. (**E**) Images from the wound-healing assay, representing the migration capacity of osteosarcoma cells. (**F**) Apoptosis of osteosarcoma cells treated with PHA-793887.

Subsequently, we examined the effects of PHA-793887 on osteosarcoma cells *in vitro*. A 3-(4,5-dimethylthiazol-2-yl)-2,5-diphenyltetrazolium bromide (MTT) assay indicated that the viability of MG63, U20S and 143B cells decreased with increasing drug concentrations (*P* < 0.05; Figure 5A). Moreover, in a colony formation assay, the PHA-793887-treated cells exhibited lower clonogenicity than the control cells in both number and size (Figure 5B, 5D).

### PHA-793887 reduces the migration of osteosarcoma cells

A scratch assay was then performed to determine the effects of PHA-793887 on the invasion and migration of osteosarcoma cells. After 24 h, the width of the scratch had clearly decreased in the control group, but had only slightly decreased in the PHA-793887 group (Figure 5C, 5E). Although the wound width decreased over time in both the control and PHA-793887 groups, the reduction was significantly greater in the control group. These results suggested that PHA-793887 reduces the migration of osteosarcoma cells.

### PHA-793887 induces apoptosis in osteosarcoma cells

We then used flow cytometry to measure the percentages of normal, necrotic, late apoptotic and early apoptotic cells after subjecting osteosarcoma cells to the control treatment or to different doses of PHA-793887 for 48 h. For controls, the respective percentages were 90.86%, 4.79%, 3.01% and 1.34%. On the other hand, for PHA-793887-treated cells, the respective percentages were 63.67%, 18.92%, 15.49% and 1.92% in the low-dose group, and 63.43%, 5.90%, 23.30% and 7.37% in the high-dose group (Figure 5F). Thus, normal cells were predominant in the control group, while apoptotic cells were predominant in the PHA-793887-treated groups.

### PHA-793887 reduces CDK1 expression in osteosarcoma cells

**Figure 6:**
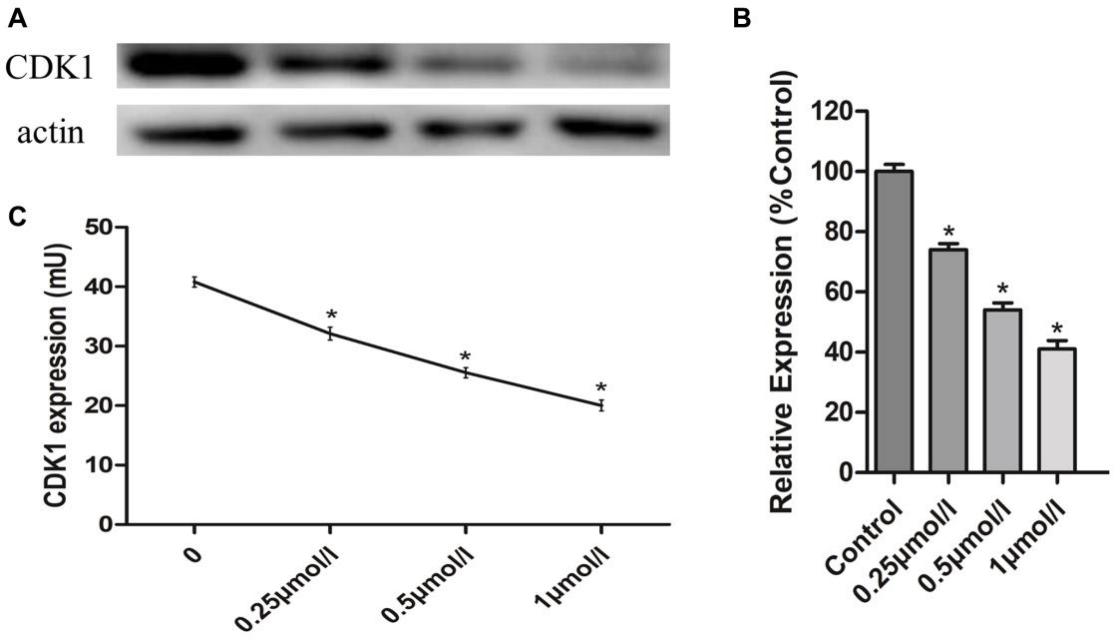
**Anti-osteosarcoma effects of PHA-793887 targeting CDK1.** (**A**) Results of western blot. (**B**) Relative expression of CDK1 (%Control) (**C**) CDK1 expression in MG63 cells.

To verify that the effects of PHA-793887 were due to its inhibition of CDK1 in osteosarcoma cells, we assessed CDK1 levels using Western blotting and an enzyme-linked immunosorbent assay (ELISA). Western blotting demonstrated that CDK1 expression decreased with increasing drug concentrations (Figure 6A, 6B). The same tendency was observed in the ELISA results (Figure 6C). These findings suggested that PHA-793887 induces apoptosis in osteosarcoma cells by inhibiting CDK1.

## DISCUSSION

Osteosarcoma is a common type of malignant bone tumor in adolescents, and is the most prevalent type of primary bone tumor. In the 1970s, the standard treatment for osteosarcoma was amputation, and the five-year survival rate was less than 20% [13]. Due to the progress in surgical techniques, the development of effective chemotherapeutic drugs and the use of adjuvant radiotherapy and chemotherapy before and after operations, limb salvage surgery has gradually replaced amputation [14] and the five-year survival rate has increased to 55–75%. However, in recent years, the treatment of osteosarcoma has encountered a bottleneck, especially for metastatic and chemotherapy-resistant cases, so new drugs and treatment strategies need to be developed based on a comprehensive understanding of the molecular mechanisms, etiology and pathogenesis of osteosarcoma.

In the present study, we identified 543 DEGs between osteosarcoma biopsy samples and normal samples, including 280 upregulated and 263 downregulated genes. These genes are potential biomarkers and treatment targets for osteosarcoma. To explore the molecular pathways contributing to the development of osteosarcoma, we then conducted GO and KEGG analyses using these DEGs. Functional and pathway enrichment analyses in Metascape revealed that the DEGs were mostly enriched in the ‘cell cycle’, ‘positive regulation of cell cycle process’, ‘DNA repair’, ‘microtubule cytoskeleton organization’ and ‘cytokinesis’. Functional and pathway enrichment analyses in DAVID indicated that the DEGs were enriched in GO terms such as the ‘positive regulation of angiogenesis’, ‘inflammatory response’, ‘heparin binding’ and ‘extracellular exosome’, and in KEGG pathways such as ‘cytokine-cytokine receptor interaction’, ‘p53 signaling pathway’, ‘HTLV-I infection’ and ‘complement and coagulation cascades’. Interestingly, the results of our GO and KEGG pathway enrichment analyses for upregulated and downregulated genes were consistent with one another. We also conducted a GSEA, which suggested that the mammalian target of rapamycin signaling pathway and autophagy were enriched in osteosarcoma patients.

Angiogenesis is a vital contributor to tumor formation and metastasis [8]. Kiyuna et al. [15] demonstrated that osteosarcoma cells promoted angiogenesis in a Nestin-driven green fluorescent protein nude mouse model, and Zhang et al. [16] reported that tetrahydrocurcumin reduced osteosarcoma cell growth by suppressing angiogenesis, suggesting that angiogenesis could be an important treatment target in osteosarcoma. Our findings were consistent with previous studies showing that cancer cells can produce and release procoagulant and fibrinolytic proteins and inflammatory cytokines [17]. Huang et al. and Song et al. demonstrated that cytokine-cytokine receptor interactions were involved in the proliferation of certain tumor cells [18–19]; thus, such interactions could also contribute to the progression of osteosarcoma.

When we constructed a PPI network from the DEGs in this study, we identified 18 hub genes with degree values ≥ 100. Hub gene expression was verified using data from GSE14359; however, these findings should be confirmed at the mRNA and protein level in patient samples in future studies. *RFC4, CDK3, CDC45* and *NCAPG* were found to be associated with osteosarcoma for the first time in our analysis, and thus could be novel diagnostic/prognostic biomarkers or treatment targets. Another hub gene detected in this study was *CDK1*, a member of the serine/threonine protein kinase family. CDK1 is a highly conserved catalytic subunit that forms protein kinase complexes with multiple interphase cyclins to promote the G2-M transition, regulate G1 progress and control the G1-S transition [20]. Thus, the upregulation of *CDK1* in osteosarcoma patients suggests that it is an important inducer of osteosarcoma and a potential treatment target.

We also functionally annotated the module genes from the PPI network, considering them to be the most important gene clusters associated with osteosarcoma. The genes in module 1 were mainly involved in ‘cell division’, ‘mitotic nuclear division’ and ‘DNA replication’. Disorder in the cell cycle and cell division can enhance the proliferation and invasion of osteosarcoma cells [21–23]. On the other hand, medical treatments have been reported to inhibit the mitotic nuclear division and replication of tumor cells [24–25]; thus, such treatments could be new therapeutic strategies for osteosarcoma.

We then performed qRT-PCR to assess the expression of five hub genes (*TOP2A, CDK1, MAD2L1, AURKA* and *RRM2*) in human normal osteoblast cells and osteosarcoma cells. These genes were all clearly upregulated in osteosarcoma cells (*P* < 0.05). We also conducted a survival curve analysis, which demonstrated that patients with lower *TOP2A* and *CDK1* levels exhibited better PFS and OS than those with higher levels of these genes (*P* < 0.05). Thus, *CDK1* and *TOP2A* expression could be used to predict the prognosis of osteosarcoma patients.

After identifying CDK1 as a potential therapeutic target in osteosarcoma, we performed a virtual screening, which demonstrated that the small molecular compound PHA-793887 could effectively inhibit CDK1 activity. A molecular docking study indicated that PHA-793887 could bind steadily to CDK1. PHA-793887 is well absorbed and is not predicted to exhibit hepatotoxicity, rodent carcinogenicity, mutagenicity or developmental toxicity potential. In previous studies, PHA-793887 has shown good efficacy in human ovarian, colon and pancreatic carcinoma xenograft models, and has been well tolerated when administered intravenously daily [26]. Several Phase I and II clinical trials have indicated that PHA-793887 is an excellent drug candidate for cancer therapy due to its potential to restore cell cycle control [27]. However, the anti-osteosarcoma activity of PHA-793887 has not been evaluated prior to this study.

In this study, we used an MTT assay, a colony formation assay, a scratch assay, flow cytometry, Western blotting and ELISA to assess the anti-osteosarcoma effects of PHA-793887 *in vitro*. In the MTT assay, the viability of MG63, U20S and 143B cells decreased with increasing doses of PHA-793887. In the colony formation assay, the clonogenicity (in both number and size) of the drug-treated cells was noticeably lower than that of the control cells, and the percentage of clone formation differed significantly according to the dose of PHA-793887. These results confirmed that PHA-793887 dose-dependently reduced the proliferation of osteosarcoma cells. The scratch assay indicated that PHA-793887 could inhibit the migration of osteosarcoma cells, while flow cytometry revealed that the percentage of apoptotic cells increased with increasing PHA-793887 doses. Our Western blotting and ELISA results demonstrated that CDK1 expression decreased with increasing drug concentrations, implying that the anti-osteosarcoma effects of PHA-793887 were due to its inhibition of CDK1. Thus, PHA-793887 dose-dependently reduced the proliferation and invasion of osteosarcoma cells by inhibiting CDK1 and inducing apoptosis.

In conclusion, we identified 543 DEGs between osteosarcoma and normal tissues in this study. GO and KEGG analyses revealed that these genes were mainly involved in the positive regulation of the cell cycle, DNA repair, cytokinesis, angiogenesis, inflammatory responses and cytokine-cytokine receptor interactions. Angiogenesis could be regarded as a treatment target in osteosarcoma. *TOP2A, CDK1, MAD2L1, AURKA* and *RRM2* were screened as hub genes in the PPI network, and patients with lower *TOP2A* and *CDK1* levels had better prognoses. PHA-793887 dose-dependently inhibited CDK1 expression and induced apoptosis in osteosarcoma cells, thus reducing their proliferation and invasion. Therefore, PHA-793887 is a promising potential drug for osteosarcoma patients.

## MATERIALS AND METHODS

### Microarray data

Gene expression data from GSE42352 were downloaded from the Gene Expression Omnibus (http://www.ncbi.nlm.nih.gov/geo) [28]. We selected 84 high-grade osteosarcoma pre-chemotherapy biopsy samples and 3 normal samples. We also downloaded gene expression data from GSE14359, including 2 normal samples and 10 osteosarcoma tissues [29]. The microarray data were subjected to background correction and standardized analysis.

### Identification of DEGs

DEGs were detected through raw data analysis using GeneSpring software (version 11.5, Agilent, CA, USA). Hierarchical clustering analysis was used to categorize osteosarcoma and normal samples. Statistically significant DEGs were identified using a classical *t*-test based on a *P*-value < 0.05 and a fold-change ≥ 2.

### GO and KEGG pathway enrichment analyses of DEGs

Metascape is a website for gene annotation, visualization and attribute characterization. We uploaded the DEGs to this website and then analyzed their GO and signal pathway enrichment. In addition, DAVID (http://david.abcc.ncifcrf.gov/) provides a comprehensive set of functional annotation tools for genes; thus, we also used DAVID for GO and KEGG pathway enrichment analyses. Further, we performed a GSEA for functional annotation and interpretation.

### PPI network construction and module selection

STRING (http://string.embl.de/) was used for PPI network analysis for bioinformatic studies. Then, hub genes and modules were screened through molecular complex detection using Cytoscape software. DAVID was used for functional and pathway enrichment analyses of the DEGs in the modules.

### Cell lines

Normal osteoblast cells (hFOB 1.19) and osteosarcoma cells (MG63, U20S and 143B) were obtained from the American Type Culture Collection. The cells were cultured in Dulbecco’s modified Eagle’s medium (DMEM, Hyclone, USA) supplemented with 10% fetal bovine serum (Gibco, MD, USA). The culture dishes were maintained in 5% CO_2_ and 95% air at 37°C.

### qRT-PCR

To verify the expression of *TOP2A, CDK1, MAD2L1,* *AURKA* and *RRM2* in osteosarcoma cell lines and normal osteoblast cells, we used FastStart Universal SYBR Green Master (ROX) mix (Roche Diagnostics) to perform qRT-PCR on a CFX96 Real-Time System (Bio-Rad) according to the manufacturer’s instructions. The mRNA levels were normalized to those of glyceraldehyde-3-phosphate dehydrogenase (*GAPDH*). The 2^-ΔΔCt^ method was used for qRT-PCR data analysis. The following primers were used: *TOP2A* sense, 5′-ACCATTGCAGCCTGTAAATGA-3′, anti-sense, 5′-GGGCGGAGCAAAATATGTTCC-3′; *CDK1* sense, 5′-AAACTACAGGTCAAGTGGTAGCC-3′, anti-sense, 5′-TCCTGCATAAGCACATCCTGA-3′; *MAD2L1* sense, 5′-GTTCTTCTCATTCGGCATCAACA-3′, anti-sense, 5′-GAGTCCGTATTTCTGCACTCG-3′; *AURKA* sense, 5′-GAGGTCCAAAACGTGTTCTCG-3′, anti-sense, 5′-ACAGGATGAGGTACACTGGTTG-3′; *RRM2* sense, 5′-CACGGAGCCGAAAACTAAAGC-3′, anti-sense, 5′-TCTGCCTTCTTATACATCTGCCA-3′.

### Clinical patient datasets and survival curve analyses

Gene expression data from 77 osteosarcoma patients (41 men and 36 women) were downloaded from Therapeutically Applicable Research to Generate Effective Treatments (https://ocg.cancer.gov/programs/target). Patients were categorized into high and low expression groups based on *TOP2A* and *CDK1* expression. PFS and OS were evaluated as prognostic outcomes.

### Crystal structure of CDK1

The ligand-binding pocket region of CDK1 was selected as the binding site for new compounds that could potentially inhibit this enzyme. Virtual filtering was performed using the LibDock module of Discovery Studio 4.5 [30]. The crystal structures of CDK1 (Protein Data Bank identifier: CY72) and 20 CDK1-targeted drugs were downloaded from the ZINC15 database (Supplementary Table 4). Protein structures were generated by removing crystalline water and other heteroatoms. The CHARMM force field and Smart Minimiser algorithm were used for energy minimization [31].

### ADME and toxicity prediction

The ADME module of Discovery Studio 4.5 was used to calculate the absorption, distribution, metabolism and excretion of selected compounds. The Toxicity Prediction by Komputer Assisted Technology (TOPKAT) module of Discovery Studio 4.5 was used to calculate the toxicity, water solubility, blood-brain barrier permeability, CYP2D6 inhibition, liver toxicity, human intestinal absorption, plasma protein binding, rodent carcinogenicity, Ames mutagenicity and developmental toxicity potential of the selected compounds.

### Molecular docking

The CDOCKER module of Discovery Studio 4.5 was used for molecular docking research. CDOCKER can produce high-precision molecular docking results based on the CHARMM field. The ligand is allowed to bend during docking, while the receptor remains rigid. For each complex posture, the CHARMM energy (interaction energy plus ligand strain) and interaction energy are used to indicate the ligand-binding affinity. During rigid and semi-flexible docking processes, crystallized water molecules are generally removed, as they may alter the formation of receptor-ligand complexes [32, 33]. Then, hydrogen atoms are added to the proteins. Different postures of each test molecule can be analyzed based on their CDOCKER interaction energy. PHA-793887 was extracted from the binding site and then realigned into the crystalline structure of CDK1 to demonstrate the reliability of the combination pattern.

### Molecular dynamics simulation

The best binding conformation for each compound/CY72 complex was chosen for the molecular dynamics simulation. The ligand/receptor complex was placed in an orthorhombic box and solvated with an explicit periodic boundary solvation water model. Sodium (ionic strength: 0.145) chloride was poured into the system to simulate the physiological environment. The system was prepared with a CHARMM force field and energy minimization (500 steps of steepest descent and 500 steps of conjugated gradient), and the resulting final root mean square gradient was 0.227. The system was slowly driven from the initial temperature (296 K) to the target temperature (320 K) in 2 ps, and equilibration simulations were performed for 8 ps. The molecular dynamics simulation (production module) was run for 30 ps with the normal pressure and temperature system (300 K). Long-range electrostatics were calculated using the particle mesh Ewald algorithm, and all bonds involving hydrogen were fixed using the linear constraint solver algorithm. The initial complex setting was selected as a reference, and the Discovery Studio 4.5 analysis trajectory protocol was used to determine the trajectory for the root-mean-square deviation, potential energy and structural characteristics.

### MTT assay

Osteosarcoma cells (MG63, U20S and 143B) were plated in 96-well culture plates at a density of 500 cells/well, and were treated with different doses of PHA-793887. MTT (Sigma, St. Louis, MO, USA) was dissolved in phosphate-buffered saline (5 mg/mL) and used to measure cell viability. On the day of measurement, the medium was replaced with fresh DMEM supplemented with 10% fetal bovine serum and diluted MTT (1:10, 10% MTT), and the cells were incubated for 3.5 h at 37°C. Then, the incubation medium was removed and the formazan crystals were dissolved in 200 μL of a dimethyl sulfoxide solution. An ELx800 absorbance microplate reader (BioTek Instruments, VT, USA) was used to quantify the reduction of MTT based on light absorbance at 570 nm.

### Colony formation assay

Osteosarcoma cells (MG63 and U20S) were seeded in Petri dishes at a density of 50 cells/cm^2^. After 24 h in culture, the cells were treated with different doses of PHA-793887. After 10 days of growth *in vitro*, the colonies were counted and characterized. Then, the colonies were rinsed with phosphate-buffered saline, fixed in 4% paraformaldehyde, stained with 5% crystal violet for 30 min and rinsed twice with water.

### *In vitro* scratch assay

MG63 cells were cultured to confluence on 24-well Permanox™ plates. A 10-μL pipette tip was used to create a consistent cell-free area in each well, and the loosened cells were washed out gently using DMEM. Then, the cells were exposed to different doses of PHA-793887. After 12 and 24 h, images of the scraped area were captured via phase contrast microscopy. The remaining wounded area and the scratch width were measured at six different points per image.

### Flow cytometry

Osteosarcoma cells (MG63) in log growth phase were seeded in six-well plates at a density of 2 × 10^5^ cells/well and treated with different doses of PHA-793887. After 48 h, the cells were harvested using Accutase detachment solution (Sigma Aldrich). Annexin-V-fluorescein isothiocyanate/propidium iodide labeling was conducted. A flow cytometer with FACSDiva Version 6.2 was used to count the stained cells.

### Western blotting

Osteosarcoma cells (MG63) in log growth phase were seeded in six-well plates at a density of 2 × 10^5^ cells/well and treated with different doses of PHA-793887. After 48 h, total proteins were harvested and electrophoretically separated. The proteins were transferred to membranes, which were treated with primary antibodies against CDK1 and β-actin and then incubated with secondary antibodies. The membranes were visualized with an enhanced chemiluminescence detection system (Pierce; Thermo Fisher Scientific, Inc.).

### ELISA

Osteosarcoma cells (MG63) in log growth phase were seeded in six-well plates at a density of 2 × 10^5^ cells/well and treated with different doses of PHA-793887. After 48 h, the ELISA was carried out and CDK1 levels were measured.

### Statistics

All data were entered into SPSS 18.0 (SPSS Inc., Chicago, IL, USA) for statistical analysis. Independent-samples *t*-tests were conducted, and *P*-values < 0.05 were considered significant.

## Supplementary Materials

Supplementary Figures

Supplementary Tables
