# Supplementary material for: Selected using bioinformatics and molecular docking analyses, PHA-793887 is effective against osteosarcoma
Source: Aging (Albany NY). 2021 Jun 21;13(12):16425–44. doi: 10.18632/aging.203165 (PMC8266349; doi:10.18632/aging.203165)
Supplement: Supplementary Figures [file aging-13-203165-s001.pdf]

SUPPLEMENTARY FIGURES

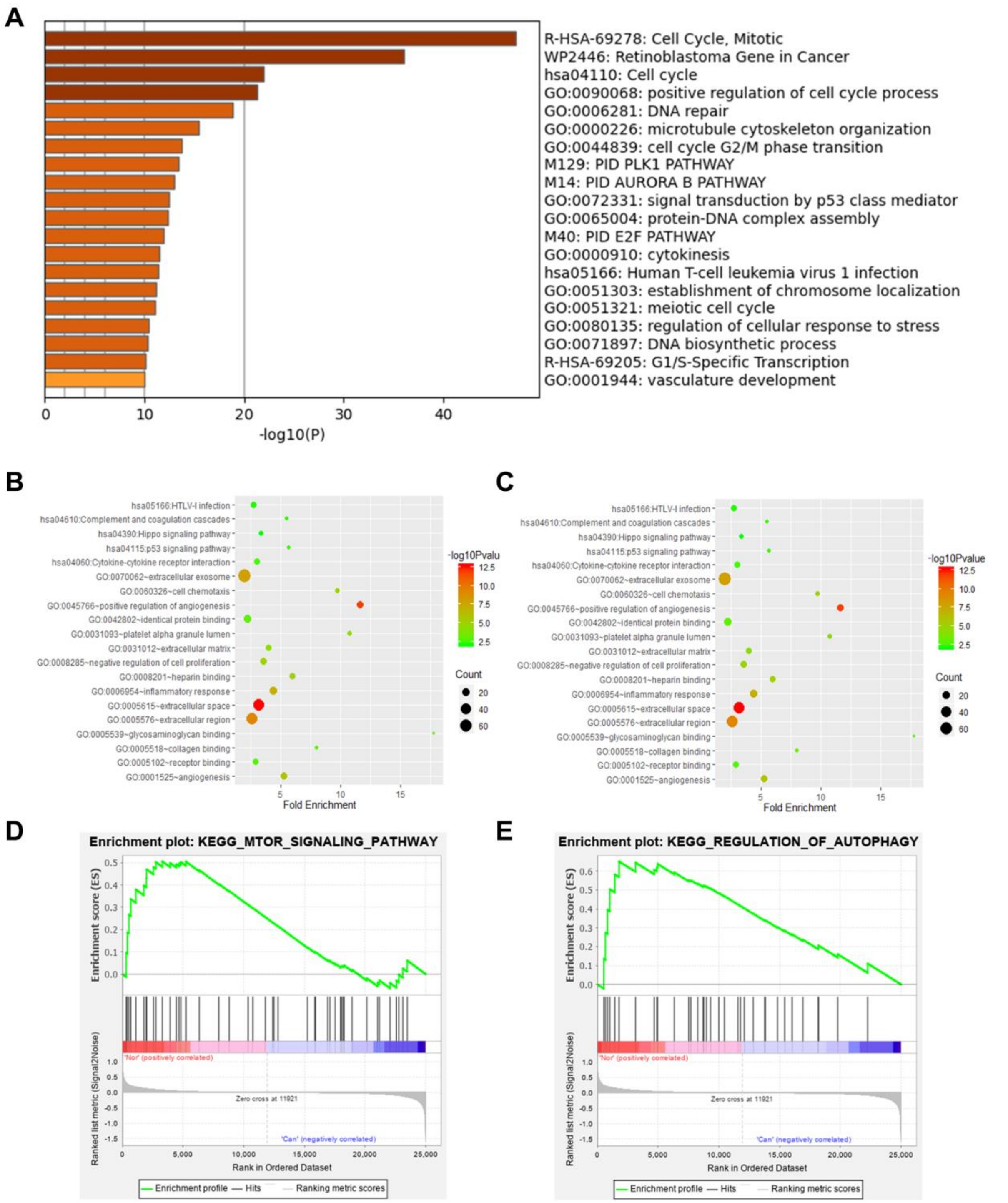

Supplementary Figure 1. Functional and pathway enrichment analyses. (**A**) GO terms and enriched KEGG pathways of the DEGs. (**B**) Functional annotation and enrichment of the upregulated genes. (**C**) Functional annotation and enrichment of the downregulated genes. (**D**, **E**) GSEA results.

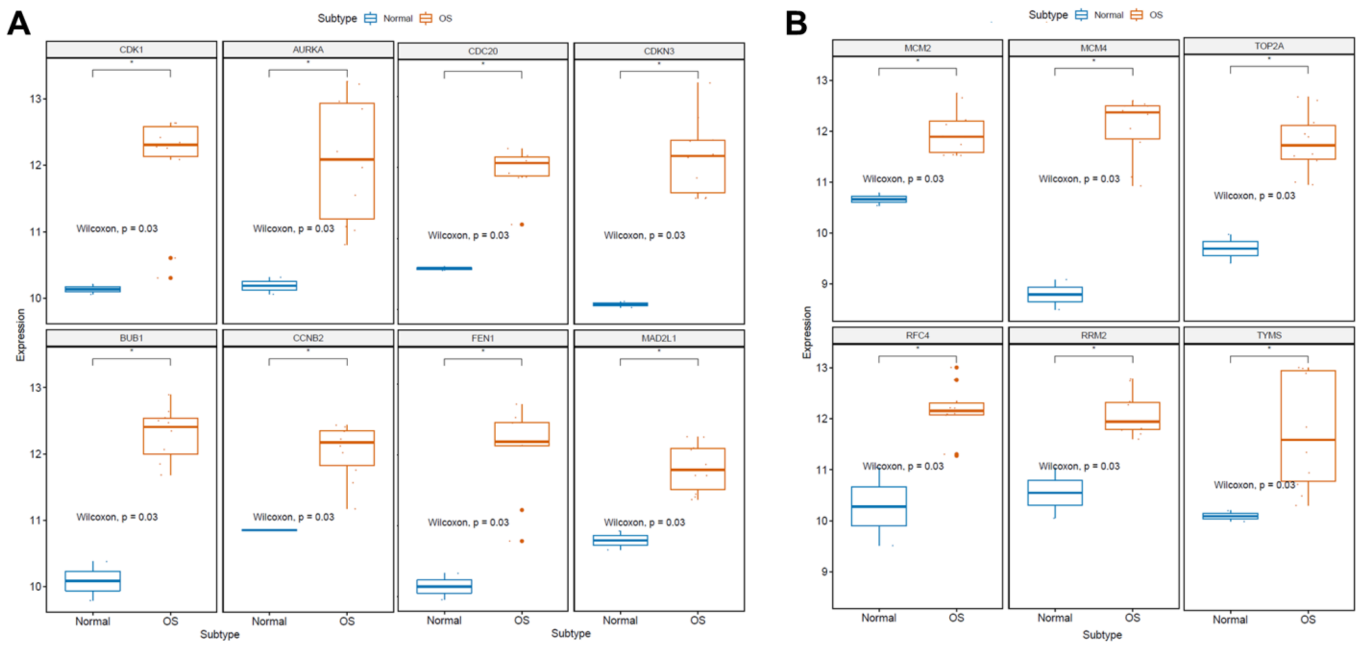

Supplementary Figure 2. The expression of hub genes in the GSE14359 dataset.
