# Supplementary material for: Selected using bioinformatics and molecular docking analyses, PHA-793887 is effective against osteosarcoma
Source: Aging (Albany NY). 2021 Jun 21;13(12):16425–44. doi: 10.18632/aging.203165 (PMC8266349; doi:10.18632/aging.203165)
Supplement: Supplementary Tables [file aging-13-203165-s002.pdf]

SUPPLEMENTARY TABLES

Supplementary Table 1. Functional and pathway enrichment analyses of the DEGs.

Expression	Category	Term	Count	%	*P* Value
up-regulated	GOTERM_BP_DIRECT	GO:0045766~positive regulation of angiogenesis	17	7.657657658	1.18E-12
	GOTERM_BP_DIRECT	GO:0006954~inflammatory response	21	9.459459459	7.45E-08
	GOTERM_BP_DIRECT	GO:0001525~angiogenesis	15	6.756756757	9.52E-07
	GOTERM_BP_DIRECT	GO:0008285~negative regulation of cell proliferation	18	8.108108108	1.19E-05
	GOTERM_BP_DIRECT	GO:0060326~cell chemotaxis	8	3.603603604	1.73E-05
	GOTERM_CC_DIRECT	GO:0005615~extracellular space	51	22.97297297	2.03E-13
	GOTERM_CC_DIRECT	GO:0005576~extracellular region	50	22.52252252	4.52E-10
	GOTERM_CC_DIRECT	GO:0070062~extracellular exosome	67	30.18018018	1.13E-08
	GOTERM_CC_DIRECT	GO:0031093~platelet alpha granule lumen	7	3.153153153	4.50E-05
	GOTERM_CC_DIRECT	GO:0031012~extracellular matrix	14	6.306306306	5.26E-05
	GOTERM_MF_DIRECT	GO:0008201~heparin binding	12	5.405405405	5.23E-06
	GOTERM_MF_DIRECT	GO:0005518~collagen binding	6	2.702702703	8.90E-04
	GOTERM_MF_DIRECT	GO:0042802~identical protein binding	21	9.459459459	0.001102044
	GOTERM_MF_DIRECT	GO:0005539~glycosaminoglycan binding	4	1.801801802	0.001349109
	GOTERM_MF_DIRECT	GO:0005102~receptor binding	13	5.855855856	0.001606711
	KEGG_PATHWAY	hsa04060:Cytokine-cytokine receptor interaction	11	4.954954955	0.003055429
	KEGG_PATHWAY	hsa04115:p53 signaling pathway	6	2.702702703	0.003804643
	KEGG_PATHWAY	hsa04610:Complement and coagulation cascades	6	2.702702703	0.004319886
	KEGG_PATHWAY	hsa05166:HTLV-I infection	11	4.954954955	0.006497285
	KEGG_PATHWAY	hsa04390:Hippo signaling pathway	8	3.603603604	0.009168069
down-regulated	GOTERM_BP_DIRECT	GO:0045766~positive regulation of angiogenesis	17	7.657657658	1.18E-12
	GOTERM_BP_DIRECT	GO:0006954~inflammatory response	21	9.459459459	7.45E-08
	GOTERM_BP_DIRECT	GO:0001525~angiogenesis	15	6.756756757	9.52E-07
	GOTERM_BP_DIRECT	GO:0008285~negative regulation of cell proliferation	18	8.108108108	1.19E-05
	GOTERM_BP_DIRECT	GO:0060326~cell chemotaxis	8	3.603603604	1.73E-05
	GOTERM_CC_DIRECT	GO:0005615~extracellular space	51	22.97297297	2.03E-13
	GOTERM_CC_DIRECT	GO:0005576~extracellular region	50	22.52252252	4.52E-10
	GOTERM_CC_DIRECT	GO:0070062~extracellular exosome	67	30.18018018	1.13E-08
	GOTERM_CC_DIRECT	GO:0031093~platelet alpha granule lumen	7	3.153153153	4.50E-05
	GOTERM_CC_DIRECT	GO:0031012~extracellular matrix	14	6.306306306	5.26E-05
	GOTERM_MF_DIRECT	GO:0008201~heparin binding	12	5.405405405	5.23E-06
	GOTERM_MF_DIRECT	GO:0005518~collagen binding	6	2.702702703	8.90E-04
	GOTERM_MF_DIRECT	GO:0042802~identical protein binding	21	9.459459459	0.001102044
	GOTERM_MF_DIRECT	GO:0005539~glycosaminoglycan binding	4	1.801801802	0.001349109
	GOTERM_MF_DIRECT	GO:0005102~receptor binding	13	5.855855856	0.001606711
	KEGG_PATHWAY	hsa04060:Cytokine-cytokine receptor interaction	11	4.954954955	0.003055429
	KEGG_PATHWAY	hsa04115:p53 signaling pathway	6	2.702702703	0.003804643
	KEGG_PATHWAY	hsa04610:Complement and coagulation cascades	6	2.702702703	0.004319886
	KEGG_PATHWAY	hsa05166:HTLV-I infection	11	4.954954955	0.006497285
	KEGG_PATHWAY	hsa04390:Hippo signaling pathway	8	3.603603604	0.009168069

Supplementary Table 2. Functional and pathway enrichment analyses of the module genes.

Module	Term	Count	*P* Value	FDR	Genes
Module 1	GO:0051301~cell division	31	3.58E-30		CKS1B, KNTC1, AURKA, PTTG1, KIF2C, CDCA8, NCAPG, BUB1, FBXO5, CCNA2, CDCA5, ERCC6L, KIF14, CDK1, KIF11, TPX2, CENPF, CDC20, CENPE, UBE2C, MCM5, SMC4, NCAPD2, FAM64A, MAD2L1, CCNB2, TIMELESS, SPAG5, ZWINT, CKS2, BUB1B
	GO:0007067~mitotic nuclear division	24	8.79E-24		CENPN, CDK1, KIF11, TPX2, KNTC1, CENPF, AURKA, CDC20, ANLN, PBK, PTTG1, AURKB, CEP55, KIF2C, FAM64A, CCNB2, TIMELESS, BUB1, FBXO5, BUB1B, CDCA5, CCNA2, ASPM, ERCC6L
	GO:0006260~DNA replication	21	1.23E-23		EXO1, GINS2, CDK1, DTL, KIAA0101, RMI2, MCM2, MCM10, RNASEH2A, MCM4, MCM5, CDT1, CDC45, RFC4, MCM7, TIMELESS, POLE2, RRM2, RRM1, CHAF1B, FEN1
Module 2	GO:0000398~mRNA splicing, via spliceosome	14	1.43E-22		SRSF1, HNRNPA3, SRSF2, SRSF5, SRSF10, MAGOH, SRRM2, ALYREF, U2AF1, SNRPB, SNRPA, SNRPD2, HNRNPA1, BUD31
	hsa03040:Spliceosome	13	1.37E-19		SRSF1, HNRNPA3, SRSF2, SRSF5, SRSF10, MAGOH, ALYREF, U2AF1, SNRPB, SNRPA, SNRPD2, HNRNPA1, BUD31
	GO:0071013~catalytic step 2 spliceosome	9	3.83E-15		SRSF1, HNRNPA3, SRRM2, MAGOH, ALYREF, U2AF1, SNRPB, SNRPD2, HNRNPA1
Module 3	GO:0002576~platelet degranulation	9	1.52E-18		ISLR, CLEC3B, SERPINE1, SERPING1, IGF2, THBS1, GAS6, SRGN, FN1
	GO:0051897~positive regulation of protein kinase B signaling	3	6.79E-04		IGF2, THBS1, GAS6
	GO:0031093~platelet alpha granule lumen	8	1.22E-17		ISLR, SERPINE1, SERPING1, IGF2, THBS1, GAS6, SRGN, FN1

Supplementary Table 3. Partial hydrogen bond interaction and π-interaction parameters for the PHA-793887/CDK1 complex.

Receptor	Compound	Interaction parameters	Donor Atom	Receptor Atom	Distances (Å)
4y72	ZINC000052509437	Hydrogen bond interaction	Molecule:H36	ASP146:OD2	2.52
			Molecule:H43	LEU83:O	2.37
			Molecule:H48	ILE10:O	2.26
		π-interaction parameters	ILE10	Molecule	4.17
			ALA31	Molecule:C26	4.13
			LEU135	Molecule	4.94
			ALA145	Molecule:C25	4.06
			Molecule:C25	VAL64	4.11
			Molecule:C25	LEU135	5.30
			Molecule:C26	VAL18	3.72
			Molecule:C26	LYS33	4.31
			PHE80	Molecule:C25	3.84
			PHE80	Molecule:C26	4.30
			Molecule	VAL18	4.87
			Molecule	LYS33	4.94

Supplementary Table 4. CDK1-targeted drugs downloaded from the ZINC15 database.

Number	Compounds	Drugs
1	ZINC000000023894	Alsterpaullone
2	ZINC000001639355	Kenpaullone
3	ZINC000002568154	5-Iodo-indirubin-3'-monoxime
4	ZINC000003814479	NU6027
5	ZINC000003924157	BMS-265246
6	ZINC000003937395	Riviciclib
7	ZINC000003938688	JNJ-7706621
8	ZINC000013983251	R547
9	ZINC000014806879	SU9516
10	ZINC000016052857	AT7519
11	ZINC000021288966	Flavopiridol
12	ZINC000028821265	Ro-3306
13	ZINC000034894449	Dinaciclib
14	ZINC000040442496	AZD-5438
15	ZINC000043128366	RGB-286638
16	ZINC000043131434	NVP-LCQ195
17	ZINC000052509437	PHA-793887
18	ZINC000053119602	Milciclib
19	ZINC000225710809	K00546
20	ZINC000261187328	NVP-2
